# Interface Trap Effect on the n-Channel GaN Schottky Barrier-Metal–Oxide Semiconductor Field-Effect Transistor for Ultraviolet Optoelectronic Integration

**DOI:** 10.3390/nano14010059

**Published:** 2023-12-25

**Authors:** Byeong-Jun Park, Han-Sol Kim, Sung-Ho Hahm

**Affiliations:** School of Electronic and Electrical Engineering, Kyungpook National University, Daegu 41566, Republic of Korea; qudwns27@knu.ac.kr (B.-J.P.); khs8465khs@naver.com (H.-S.K.)

**Keywords:** gallium nitride, Schottky barrier-MOSFET, UV optoelectronics, interface defect

## Abstract

Ultraviolet (UV) photodetectors are key devices required in the industrial, military, space, environmental, and biological fields. The Schottky barrier (SB)-MOSFET, with its high hole and electron barrier, and given its extremely low dark current, has broad development prospects in the optoelectronics field. We analyze the effects of trap states on the output characteristics of an inversion mode n-channel GaN SB-MOSFET using TCAD simulations. At the oxide/GaN interface below the gate, it was demonstrated that shallow donor-like traps were responsible for degrading the subthreshold swing (SS) and off-state current density (I_off_), while deep donor-like traps below the Fermi energy level were insignificant. In addition, shallow acceptor-like traps shifted the threshold voltage (V_t_) positively and deteriorated the SS and on-state current density (I_on_), while deep acceptor-like traps acted on a fixed charge. The output characteristics of the GaN SB-MOSFET were related to the resistive GaN path and the tunneling rate due to the traps at the metal (source, drain)/GaN interface. For the UV responses, the main mechanism for the negative V_t_ shift and the increases in the I_on_ and spectral responsivity was related to the photo-gating effect caused by light-generated holes trapped in the shallow trap states. These results will provide insights for UV detection technology and for a high-performance monolithic integration of the GaN SB-MOSFET.

## 1. Introduction

The ability to characterize ultraviolet (UV) emission spectra is important because it can provide important insights into the semiconductor industry, military aircraft survivability equipment, space-based communication, solar science, and even biological agents [1,2,3,4,5,6,7,8,9,10,11]. Within the semiconductor industry, extreme UV (EUV) lithography technology using a wavelength of 13.5 nm has facilitated the creation of a complex integrated circuit fabrication process for the most advanced microchips (7, 5, and 3 nm modes). Moreover, EUV detectors are the key components used for monitoring and calibrating photon beam intensity [4,5,6]. In the military field, missile warning receivers (MWRs) using UV signal detection have been studied to counter missile systems such as man-portable air defense systems (MANPADS) [8,9]. In these systems, spectra from 240 to 280 nm are applied to detect the tail flames of approaching missiles. In addition, UV reflection monitoring technologies, such as spaceborne imagers (e.g., satellites) and unmanned aerial vehicles (UAVs), are important components for the tracking and managing of missiles, aircraft, and the Earth’s resources [11]. Accordingly, there is increasing demand for advanced-performance UV photodetectors that can detect light in the UV spectral region and for the monolithic integration of UV optoelectronic devices.

Materials related to gallium nitride (GaN), which have a direct wide bandgap, are excellent candidates for UV photodetectors due to their excellent solar blindness, high quantum efficiency, and superior thermal and chemical stability [12,13,14,15]. Ternary alloys, such as aluminum gallium nitride (Al_x_Ga_1−x_N), are suitable semiconductors for UV detection applications because Al_x_Ga_1−x_N has an energy gap that is tunable from 3.4 to 6.2 eV depending on the Al mole fraction. Moreover, the cut-off wavelength according to the Al mole fraction of AlGaN can be controlled from 365 to 200 nm [16].

Among the various types of GaN-based UV photodetectors, the metal–semiconductor–metal (MSM) type is an attractive candidate because of its very simple fabrication process and high UV-to-visible rejection ratio (UVRR) due to the back-to-back Schottky barrier structure. In addition, Schottky barrier (SB)-MOSFETs have the same structure as MSM-type photodetectors (except for the gate region), meaning that they can be easily integrated at both the circuit and system levels. In our recent study, we demonstrated the operation of a hybrid GaN UV active pixel sensor (APS) with an Si CMOS APS controller and a GaN UV passive pixel sensor (PPS) using a GaN SB-MOSFET, both of which applied MSM-type UV photodetectors [17,18]. However, the sensing performance of these fabricated APS and PPS devices exhibited a high dark current, low responsivity, and poor UVRR, which was attributed to their low-quality epitaxial layer and high defect density. A high dark current caused by trap-assisted leakage through defects deteriorates the photo-response characteristics of UV photodetectors, resulting in poor detection capability [19,20,21,22]. Moreover, during imaging processes, the high noise (fixed pattern and temporal) caused by the defects decreases the signal-to-noise ratio (SNR), adversely affecting the quality of the sensing image [11,22,23,24,25,26,27]. Moreover, since these defects result in current collapse and poor breakdown voltages in GaN-based power semiconductors, reducing both the defects and the dark current are essential challenges to be addressed. Ultimately, it is necessary to investigate the causality of defects and interface traps quantitatively to achieve high-performance optoelectronic integrated circuits, such as UV pixel sensors. Owing to their high hole and electron barrier due to the p-type GaN, and given their extremely low dark current and simple fabrication process, inversion mode n-channel GaN SB-MOSFETs are expected to become promising candidates for use as UV photodetectors.

Based on the reported information about traps in GaN materials, we conducted a simulation to examine the effects of both donor- and acceptor-like trap states at the GaN interface, including the quantitative values of defects. We also analyzed the electrical characteristics of inversion mode n-channel SB-MOSFETs according to the interface trap state levels moving from the band edge to the midgap. By adjusting the defect density in the simulation, the output current (I_D_) was closely matched to the measured data from published work, providing insights into the interface defects distributed in fabricated devices. For UV optoelectronics, it was confirmed that the V_t_ shift and spectral responsivity were related to the photo-gating effect caused by light-generated holes trapped in the shallow states.

## 2. Device Simulation Methodology

### 2.1. Device Structure and Simulation Models

**Figure 1:**
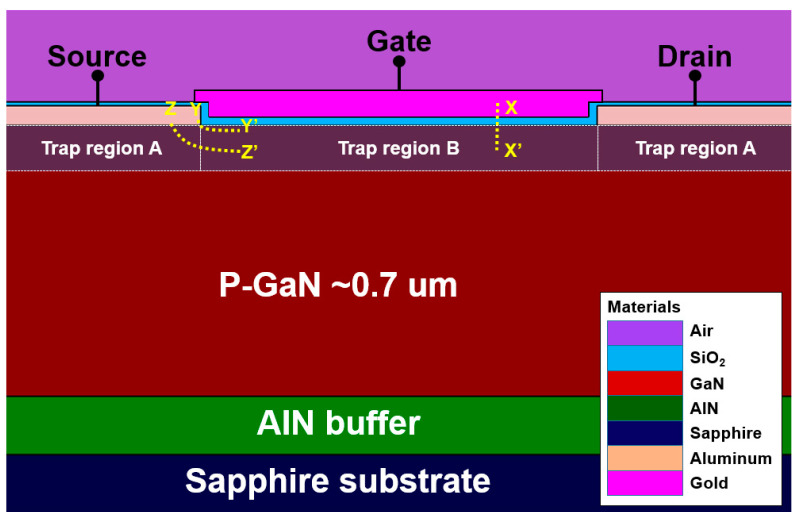
Schematic cross-sectional view of a simulated inversion mode n-channel GaN SB-MOSFET (directly extracted from the TCAD simulation results).

Figure 1 display two-dimensional view of a simulated inversion mode n-channel GaN Schottky barrier (SB)-MOSFET, which can be applied as UV photodetectors and switching transistors in image sensors [28,29]. All the device parameters of the GaN SB-MOSFET reported in [28] were repeated for this simulation study. The carrier concentration of the bulk p-type GaN used in the simulation was set to 2.7 × 10^17^ cm^−3^, which was slightly lower than the reported value of 8.6 × 10^17^ cm^−3^. The trap regions A and B refer to the different models of the trap state distribution. In the Silvaco Atlas 2D simulation, the distribution of the trap states is defined using two models: discrete trap state distribution and Gaussian trap state distribution. In this simulation study, the discrete trap state distribution model was used for trap states under the metal (source, drain)/GaN interface, and the Gaussian trap state distribution model was employed for trap states under the SiO_2_/GaN interface [30]. Table 1 lists the device parameters of the reported and simulated inversion mode n-channel GaN SB-MOSFETs used in the Silvaco Atlas 2D simulator.

The Silvaco Atlas 2D simulations were performed using the universal Schottky tunneling model (UST) and the tunneling probability was determined using the WKB approximation. In this model, the tunneling current is represented by the localized tunneling rates at grid locations near the Schottky contact. In addition, the thermionic emission model, image force barrier lowering model, Shockley–Read–Hall (SRH) recombination, Auger recombination, the field-dependent mobility model, and the inversion layer mobility model reported by Lombadi were employed [30].

### 2.2. Defect Models

Most of the interface traps were experimentally identified using the DLTS/DLOS method. In this simulation study, two types of interface trap states present in the observed GaN were studied. First, donor-like trap states were considered, which originated from the nitrogen vacancies (V_N_) and interstitial carbon (C_I_). The formation energies of the V_N_ have been reported as shallow donor levels of 0.06 eV [31] and 0.25 eV [32] from the conduction band edge. Moreover, during decomposition and reformation in GaN epitaxial growth at temperatures of 600–1100 °C, nitrogen dissociated from the GaN surface and formed N_2_ and NH_3_ molecules, resulting in a nitrogen-segregated surface that could be a source of V_N_ [33,34,35]. In contrast, the C_I_ was related to dislocations and/or residual carbon impurities that occurred during epitaxial growth, which manifested as deep donor behavior in p-type GaN [36,37,38]. These states were reported to be shallow donor levels with energies of 1.28 [36] and 1.35 eV [37] from the conduction band edge. Second, acceptor-like trap states were considered that originated from gallium vacancies (V_Ga_), residual Mg_Ga_, residual C, and substitutional carbon (C_N_) [33,37,39]. Trap states located at E_c_−1.5 eV have been employed in various GaN-based device [40,41,42,43]. The positive interface fixed charge density (N_f_) was considered as 2.2 × 10^12^ cm^−2^. It has also been reported that the Ga-face of GaN crystals has a positive surface polarization charge of 10^13^ cm^−2^, while the N-face of GaN has a negative value [44,45,46].

**Figure 2:**
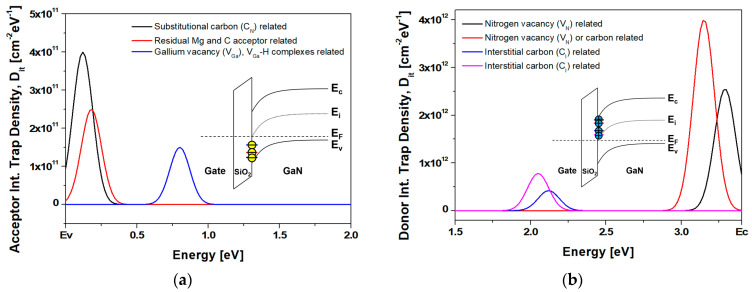
(**a**) Acceptor-like interface trap state density and (**b**) donor-like interface trap state density defined for the Gaussian distribution used at the SiO_2_/GaN interface.

As depicted in Figure 1 and Figure 2, and in Table 2, the donor- and acceptor-like trap states were defined as a defect model using the Gaussian distribution in the SiO_2_/GaN interface and a trap model using the discrete trap level at the metal/GaN interface [30,47,48]. Table 2 presents a summary of the trap energy positions and origins of the trap states in the c-plane GaN applied in this simulation study.

**Figure 3:**
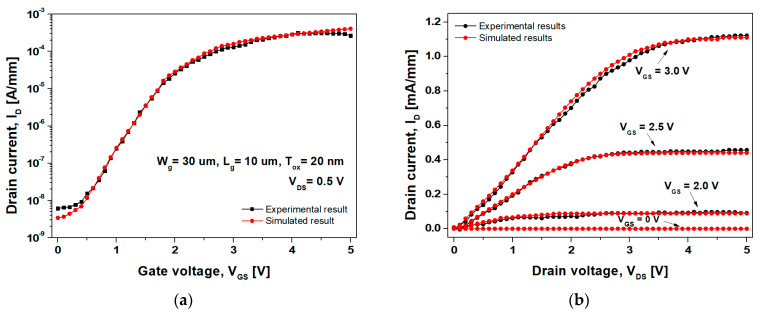
(**a**) Transfer and (**b**) output characteristics of the simulated and fabricated inversion mode n-channel GaN SB-MOSFETs.

Figure 3a displays the simulated and experimental transfer curves of the inversion mode n-channel GaN SB-MOSFET achieved using the combinations of interface traps listed in Table 2. Figure 3b displays the simulated and experimental output characteristics, which established the validity of the simulation used in this study.

### 2.3. Effect of Interface Trap States

Interface traps modify the localized electric field and energy band profiles, and they change the barrier parameters. The ionization probabilities of the trap state are expressed as follows [49]:

(1) $$N_{TD}^{+}=N_{TD}/\left(1+g_{D}e^{\frac{E_{F}-E_{T}}{kT}}\right),$$

(2) $$N_{TA}^{-}=N_{TA}/\left(1+g_{A}e^{\frac{E_{T}-E_{F}}{kT}}\right),$$

where $N_{TD}^{+}$ and $N_{TA}^{-}$ are the numbers of ionized interface donor- and acceptor-like traps, $N_{TD}$ and $N_{TA}$ are the total interface trap state densities, $g_{D}$ and $g_{A}$ are the degeneracy factors, and $E_{T}$ and $E_{F}$ are the trap and Fermi energy levels, respectively.

**Figure 4:**
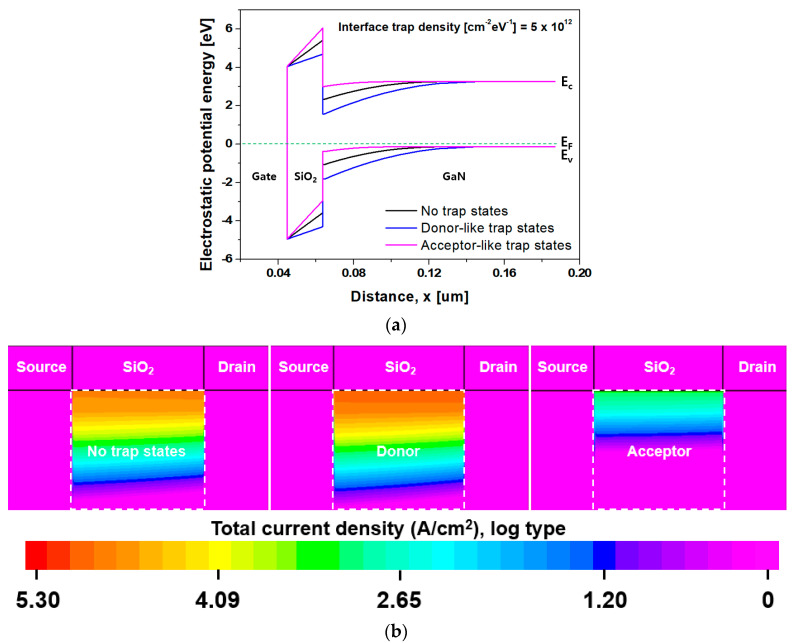
(**a**) Energy band diagram at the MOS interface along the cutline of X−X’ of Figure 1 under the zero-bias condition; and (**b**) contour map of the total current density at a V_GS_ of 4.0 V and V_DS_ of 1.0 V according to the type of trap state at the SiO_2_/GaN interface.

Figure 4a shows the energy band diagram of the simulated inversion mode n-channel GaN SB-MOSFET extracted along the cutline of X−X’ of Figure 1. Figure 4b displays a contour map of the current density at a V_GS_ of 4.0 V and a V_DS_ of 1.0 V according to the type of trap state at the SiO_2_/GaN interface, where both the donor- and acceptor-like trap state densities were 5.0 × 10^12^ cm^−2^eV^−1^. The current densities according to the no-trap state condition, donor-like trap states, and acceptor-like trap states were determined as 9.7 × 10^4^, 1.2 × 10^5^, and 1.5 × 10^3^ A/cm^2^, respectively. The SiO_2_/GaN interface traps changed the electron concentration in the channel, which significantly affected the I_off_, on-state current (I_on_), V_t_, SS, R_on_, and BV of the MOSFETs.

**Figure 5:**
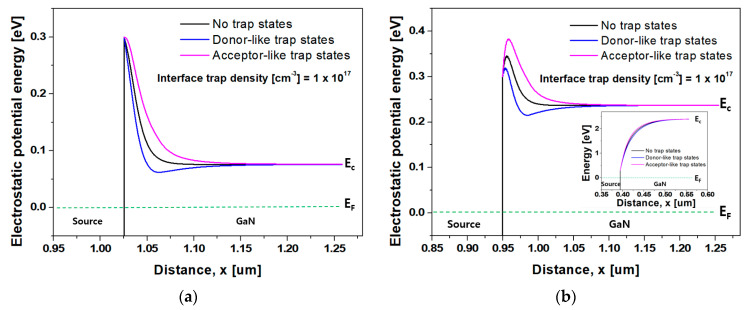

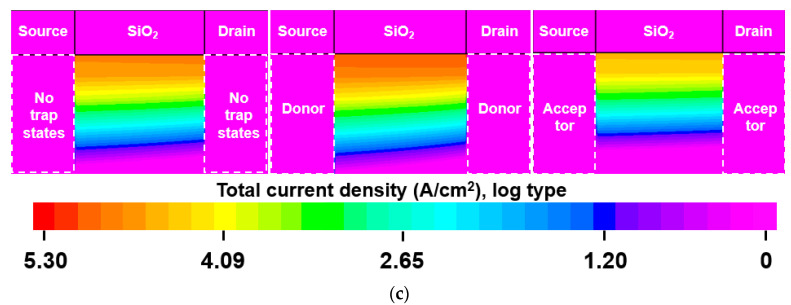
(**a**) Energy band diagram at the metal/GaN interface along the cutline of Y−Y’ (which has a strong influence on the gate bias voltage) at a V_GS_ of 4.0 V in Figure 1; (**b**) metal/GaN interface along the cutline of Z−Z’ (which has a weak influence on the gate bias voltage) at a V_GS_ of 4.0 V in Figure 1. Inset is an energy band diagram of the metal/GaN interface under the zero-bias condition; and (**c**) contour map of the total current density at a V_GS_ of 4.0 V and V_DS_ of 1.0 V according to the type of trap state at the metal/GaN interface.

As displayed in Figure 5a,b, energy band diagrams at the metal/GaN interface were extracted along the cutlines of Y−Y’ and Z−Z’ at a V_GS_ of 4.0 V. The energy band diagram corresponding to the cutlines of Y−Y’ and Z−Z’ means that variations in the Schottky barrier height and depletion width due to the trap states are affected by the gate bias voltage. Owing to band bending by the gate bias voltage, the barrier height and depletion width decrease as the metal/GaN interface approaches closer to the gate electrode.

Figure 5a,b demonstrate that the Schottky barrier height and the depletion width changed depending on the ionized traps at the metal/GaN interface and band bending by the gate bias voltage. This resulted in changes in the Schottky barrier tunneling probability and, consequently, the tunneling current (J_T_) due to thermionic field emissions (TFEs) [50,51]. Figure 5c displays a contour map of the current density at a V_GS_ of 4.0 V and a V_DS_ of 1.0 V according to the type of trap state at the metal/GaN interface. The donor- and acceptor-like trap state densities were set to 1.0 × 10^17^ cm^−3^. The current densities according to the no-trap state condition, donor-like trap states, and acceptor-like trap states were revealed as 9.7 × 10^4^, 1.5 × 10^5^, and 4.4 × 10^4^ A/cm^2^, respectively. As shown in Figure 5, movement of electrons supplied from the source is expected to be the dominant cutline of Y−Y’.

## 3. Results and Discussion

### 3.1. Static Characteristics of the GaN SB-MOSFET

**Figure 6:**
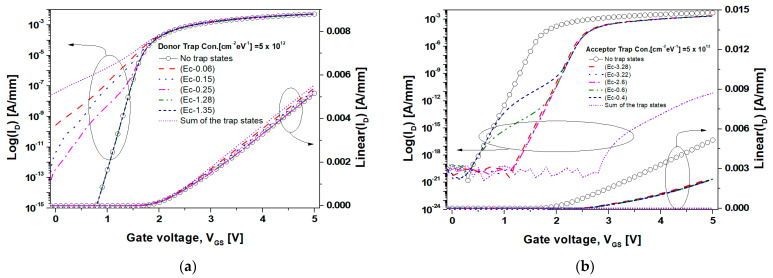
Transfer I_D_−V_GS_ characteristics of the inversion mode n-channel GaN SB-MOSFET at a V_DS_ of 1.0 V according to the trap state levels: (**a**) donor- and (**b**) acceptor-like trap states were defined at the SiO_2_/GaN interface.

Figure 6a,b display the transfer characteristics of the inversion mode n-channel GaN SB-MOSFETs as the SiO_2_/GaN interface trap state levels move from the band edge to the midgap. The concentration of donor- and acceptor-like traps was set to 5.0 × 10^12^ cm^−2^eV^−1^, and the captured cross-sections of the electrons and holes were set to 1.1 × 10^−15^ cm^−2^.

As displayed in Figure 6a, the I_off_ for the GaN SB-MOSFET without trap states was 2.6 × 10^−21^ A/mm, although it increased significantly to 2.6 × 10^−10^ and 3.1 × 10^−13^ A/mm when the donor-like traps of (E_c_−0.06) and (E_c_−0.25) associated with the V_N_ were distributed, respectively. This result indicated that a large number of shallow donor-like trap states had already ionized under the zero-bias condition because the (E_F_−E_T_) was sufficiently low to ionize most of the trap states, as demonstrated in Equation (1). The positive ionized donor-like traps increased the electric potential of the SiO_2_/GaN interface and accumulated electrons in the channel. Owing to this high electron concentration in the channel, the depletion regions of both the source and drain ends of the channel were further reduced, resulting in the barrier tunneling, as indicated in Figure 5a.

The subthreshold swings (SSs) in the shallow trap conditions of (E_c_−0.06) and (E_c_−0.25) were 358 and 227 mV/decade, respectively. The positive gate bias (V_GS_) shifted the trap energy level closer to the Fermi energy level, which modified the ionization rate of the donor-like trap states, which resulted in increases in the interface capacitance (C_it_) and SS. In comparison, the I_off_ and SS for the deep donor-like trap states of (E_c_−1.28) and (E_c_−1.35) were unchanged when compared to that of the GaN SB-MOSFET without trap states. Importantly, since the (E_c_−E_F_) of the SiO_2_/GaN interface in this simulation study was 1.09 eV, the trap states of (E_c_−1.28) and (E_c_−1.35) located below the Fermi energy level resulted in a high probability of being filled with electrons, causing a poor number of ionized donor-like traps. Unlike Figure 6b, the V_t_ and I_on_ barely altered, which was attributed to the reduced ionization as the conduction band approached the Fermi energy level due to the positive V_GS_. From the simulation results, we concluded that the shallow trap-related nitrogen vacancy (V_N_) shall be carefully reduced in the top epitaxial layer of the MOSFET.

As displayed in Figure 6b, the I_on_ of the GaN SB-MOSFET without trap states at a V_GS_ of 5.0 V was 5.16 mA/mm, although this decreased to 2.32 mA/mm when the acceptor-like trap states were distributed. In particular, the I_on_ of the GaN SB-MOSFET in consideration of all the acceptor-like trap states was 7.2 × 10^−12^ mA/mm, which was an order of magnitude of approximately 10^−12^ lower than for the GaN-SB MOSFET without the trap states. This result indicated that the negative charge property corresponding to the sum of the ionized acceptor-like traps significantly reduced the electric potential at the SiO_2_/GaN interface, resulting in an increase in the V_t_. The ionized acceptor-like traps would enhance impurity ion scattering and channel resistance, while electrons accumulated due to ionized donor-like traps screened the electric field associated with the positive charge of the ionized donor-like traps at the interface. Furthermore, due to the low electron concentration of the channel, the depletion width near the Schottky interface expanded. This resulted in enhanced relaxation of the electric field near the Schottky interface, causing the low barrier tunneling probability and I_on_. The V_t_ of the GaN SB-MOSFET with an acceptor-like trap state density of 5.0 × 10^12^ cm^−2^eV^−1^ was 2.5 V, representing an increase compared with the value of 1.7 V without trap states. In the subthreshold region, the acceptor-like trap state exhibited a different inversion process as it moved from a deep to a shallow trap state. The trap states of (E_c_−3.28), (E_c_−3.22), and (E_c_−2.6) were distributed deeply below the Fermi energy level under a zero-bias condition, which resulted in an ionization probability of 1.0. This indicated that there was no deterioration in the SS because the charge variation of the ionized acceptor-like trap according to the positive V_GS_ was insignificant. Moreover, the trap states of (E_c_−0.6) and (E_c_−0.4) were distributed above the Fermi energy level under a zero-bias condition. When the V_GS_ was applied, each trap state level sequentially approached the Fermi energy level, causing a high ionization probability. Owing to the charge variation caused by the generation of the ionized acceptor-like traps at the V_GS_ values of 0.56 and 0.78 V, as displayed in Figure 6b, the SS increased to 273 mV/decade. This result indicated a deterioration compared with the value of 65 mV/decade without trap states. Hence, this affected the device’s switching performance significantly, while the donor-like traps had no significant effect on the device’s on-state performance due to their neutral-only behavior according to the positive V_GS_. In addition, the trap densities of the shallower levels (such as V_N_) and the dislocation-related point defects due to the poor epitaxial growth should initially be reduced for improved switching performance.

**Figure 7:**
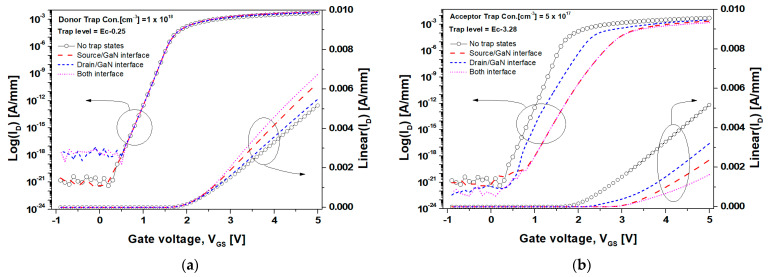
Transfer I_D_-V_GS_ characteristics of the inversion mode n-channel GaN SB-MOSFET under a V_DS_ of 1.0 V according to the trap state region: (**a**) donor- and (**b**) acceptor-like trap states were defined at the metal/GaN interface.

**Figure 8:**
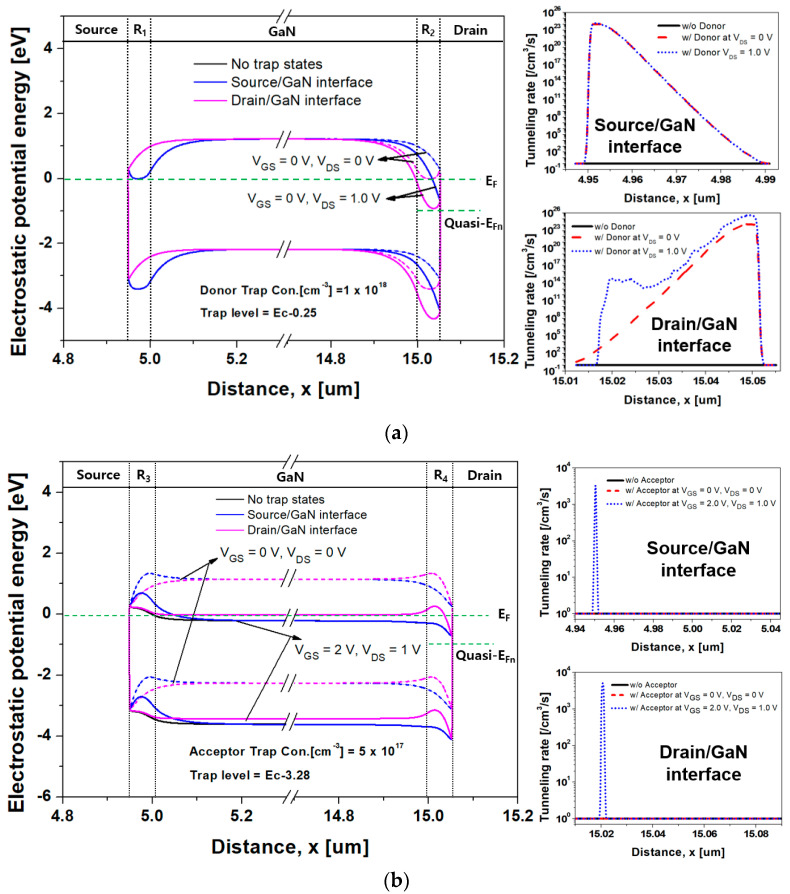
Energy band diagram and tunneling rate of the simulated inversion mode n-channel GaN SB-MOSFET from source to drain: (**a**) with and without donor-like trap states and (**b**) with and without acceptor-like trap states at the metal/GaN interface according to the applied bias voltage.

Figure 7 displays the transfer characteristics of the GaN SB-MOSFET according to the donor- and acceptor-like trap states at the metal/GaN interface. Figure 8 also displays the energy band diagram and tunneling rate of the GaN SB-MOSFET from the source to drain according to the trap state at the metal/GaN interface, as extracted using a TCAD simulation. In Figure 7a, the I_off_ of the GaN SB-MOSFET with donor-like trap states at the drain/GaN interface was 9.2 × 10^−19^ A/mm, indicating a substantive reduction compared with the value of 2.6 × 10^−21^ A/mm with donor-like trap states at the source/GaN interface. As displayed in Figure 8a, the ionized donor-like traps accumulated electrons near the Schottky interface, reduced the depletion region, and fully connected the channel to the donor-like trap region. This resulted in an increase in the tunneling rate and a decrease in the series resistance (R_1_ and R_2_) between the source/drain end of the channel and the source/drain electrode [52]. Fortunately, when the ionized donor-like traps were distributed only at the source/GaN interface, electrons supplied from the source were blocked by the high barrier of the p-type GaN. In comparison, the ionized donor-like traps distributed on the drain/GaN interface reduced the R_2_ and decreased the high resistive carrier path from the channel to the drain contact, meaning electrons could pass more easily through the drain contact at a V_DS_ of 1.0 V. Moreover, when a V_DS_ of 1.0 V was applied, as the electric field increased in the drain/GaN interface, the J_T_ through the TFE increased significantly. In addition, the thermionic emission current (J_th_) remained approximately constant, resulting in an increased tunneling rate at the drain/GaN interface. This off-state performance would significantly degrade the dark current and photo-response characteristics, resulting in poor sensing performance. The I_on_ of the GaN SB-MOSFET due to the ionized donor-like traps on the drain/GaN interface at a V_GS_ of 5.0 V was 5.5 mA/mm. This increased to 6.3 mA/mm considering the ionized donor-like traps of the source/GaN interface, which indicated that the on-state current of the GaN SB-MOSFET was dominated by the tunneling current supplied from the source contact.

As demonstrated in Figure 7b, the I_off_ of the GaN SB-MOSFET due to the acceptor-like trap states at the drain/GaN interface was 2.6 × 10^−22^ A/mm, which was slightly lower than the value of 8.8 × 10^−22^ A/mm due to trap states at the source/GaN interface. This result was attributed to the barrier height and depletion width due to the ionized acceptor-like traps at the drain/GaN interface. Moreover, the ionized acceptor-like traps established a high series resistance (R_3_ and R_4_). The effective high resistive carrier path from the channel to the source/drain electrode was longer when the ionized acceptor-like traps was distributed at the metal/GaN interface, as displayed in Figure 8b, which resulted in an decrease in the tunneling rate. At a V_GS_ = 2.0 V and a V_DS_ = 1.0 V, the I_on_ of the GaN SB-MOSFET due to the acceptor-like trap states at the source/GaN interface and drain/GaN interface were 1.8 × 10^−10^ A/mm and 5.2 × 10^−7^ A/mm, respectively. This result indicated that the high barrier due to the ionized acceptor-like traps at the source/GaN interface blocked the movement of electrons supplied from the source, significantly reducing the number of electrons contributing to the tunneling current compared to the barrier at the drain/GaN interface. We carefully concluded that the formation of a high-quality interface and barrier on the drain side would reduce the dark current caused by current leakage.

### 3.2. Output Characteristics Related to the Acceptor-like Trap State

**Figure 9:**
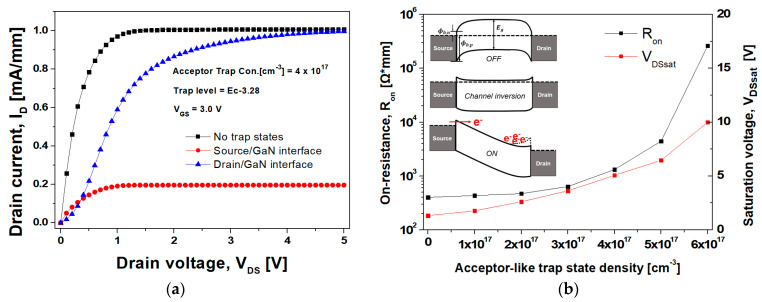
Output characteristics of the GaN SB-MOSFET due to the acceptor-like trap states: (**a**) variation in the output current according to the metal/GaN interface trap region; (**b**) R_on_ and V_DSsat_ variations for the different acceptor-like trap state densities at the drain/GaN interface. The inset displays the schematic energy band diagrams from source to drain for the three operation modes.

Figure 9 displays the output characteristics of the GaN SB-MOSFET related to the acceptor-like trap states at the metal/GaN interface. The inset in the figure displays the energy band diagram from source to drain for the three operation modes of the GaN SB-MOSFET. As depicted in Figure 9a, at a V_DS_ of 5.0 V, the saturation current density (I_Dsat_) of the GaN SB-MOSFET due to the acceptor-like trap states at the source/GaN interface was 0.2 mA/mm, which was significantly lower than the value of 1.0 mA/mm without the trap states.

The saturation voltage (V_DSsat_) of the GaN SB-MOSFET due to the acceptor-like trap states at the drain/GaN interface shifted from 1.32 to 5.0 V, while the I_Dsat_ of the GaN SB-MOSFET was 0.9 mA/mm. This was attributed to electrons accumulated at the drain end of the channel. The current saturation resulted in the increase in the electric field from the source to drain slowing down due to the channel debiasing at a high V_DS_. Eventually, the electric field did not increase any further, resulting in the velocity saturation of the electrons extracted from the source. Under this condition, a depletion region formed at the drain end of the channel, meaning that the electron concentration dropped below the acceptor concentration in the p-type GaN. As displayed in the inset of Figure 9b, during the on-state of the high V_DS_, the Schottky barrier height and depletion width formed by the ionized acceptor-like traps accumulated some of the electrons moving from the source to the drain. Hence, the drain end of the channel was not depleted, resulting in no electric field saturation from the source. The Schottky barrier height and depletion width of the drain side due to the ionized acceptor-like traps increased both the R_on_ and V_DSsat_ of the GaN SB-MOSFET, while the effect of the ionized donor-like traps on the drain side was insignificant.

### 3.3. Photo-Response Characteristics of SB-MOSFET

Conventionally, active pixel UV image sensors are 3T APS and 4T APS through a CMOS process [53,54]. The pixel circuitry of these types of devices consists of a reset transistor, a source follower (i.e., a buffer), a switching transistor, and a photodetector. Among the reported APS circuitries, Hong et al. achieved high APS sensitivity by adopting a phototransistor as a photodetector [29]. However, their APS suffered from low spectral responsivity (R_ph_) and a poor signal-to-noise ratio (SNR) caused by the dark current (I_dark_) due to the unintended tunneling and SRH generation [25,27]. Moreover, current leakage associated with defects in the transistor can be responsible for a high I_dark_.

**Figure 10:**
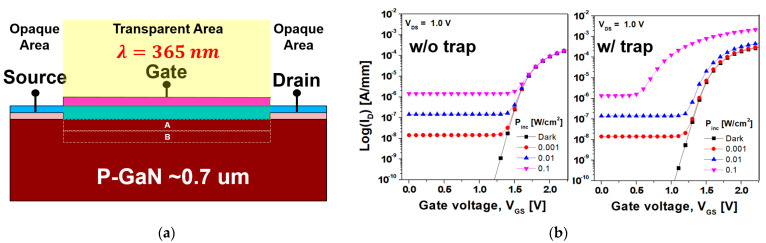

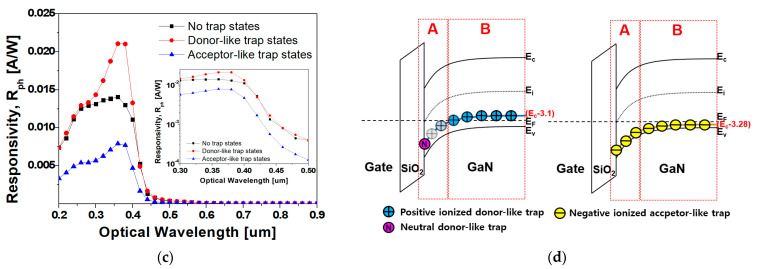
(**a**) Schematic of the UV irradiation for the GaN SB-MOSFET. (**b**) Photo-induced transfer characteristics of the GaN SB-MOSFET with (right) and without (left) donor-like trap states at a V_DS_ = 1.0 V. (**c**) R_ph_ of GaN SB-MOSFET according to type of the trap state. (**d**) Schematic energy band diagram of changes in the behavior of ionized donor- (left) and acceptor-like traps (right) according to difference between the Fermi energy level and trap state level across the MOS interface.

Figure 10 displays the availability of the GaN SB-MOSFET as a phototransistor and the effect of the photo response characteristics according to the type of the trap state. In this simulation study, a GaN SB-MOSFET composed of a transparent top gate and an opaque source/drain was employed, as displayed in Figure 10a. The traps were distributed at the SiO_2_/GaN interface and the bulk GaN area, and the concentration of the donor- and acceptor-like trap states was set to 10^18^ cm^−3^. The trap energy levels of the donor- and acceptor-like trap state were set to (E_c_−3.1) and (E_c_−3.28), respectively. Areas A and B refer to areas near the SiO_2_/GaN interface and slightly away from the SiO_2_/GaN interface, respectively. Figure 10b displays the photo-induced transfer characteristics of the GaN SB-MOSFET with and without the donor-like trap states at incident power densities (P_inc_) ranging from 0.001 to 0.1 W/cm^2^. Under UV irradiation, the conductivity of the channel increased due to the generated electron–hole pairs (EHPs), and this photoconductive (PC) effect resulted in an increase in the I_off_ of the GaN SB-MOSFET. The I_off_ of the GaN SB-MOSFET without trap states under dark conditions was 2.5 × 10^−21^ A/mm, which increased to 1.4 × 10^−8^, 1.3 × 10^−7^, and 1.3 × 10^−6^ A/mm for P_inc_ values of 0.001, 0.01, and 0.1 W/cm^2^, respectively, during UV irradiation.

Under the dark condition of a V_GS_ = 3.0 V, the I_on_ of the GaN SB-MOSFET with donor-like trap states was 5.5 × 10^−4^ A/mm, indicating an increase compared with the value of 4.3 × 10^−4^ A/mm without trap states. This result was attributed to the positively ionized donor-like traps in Areas A and B, except for the SiO_2_/GaN interface. Under UV irradiation with a V_GS_ = 3.0 V, the I_on_ of the GaN SB-MOSFET with donor-like trap states were 5.9 × 10^−4^, 9.1 × 10^−4^, and 3.4 × 10^−3^ A/mm at P_inc_ values ranging from 0.001 to 0.1 W/cm^2^, which indicated an increase compared with the value of 4.3 × 10^−4^ A/mm without trap states. This result indicated that light-generated holes in Areas A and B were captured in donor-like trap states located near the valence band, resulting in the accumulation of more positively ionized donor-like traps and a reduction in the conduction band (i.e., the photo-gating [PG] effect). Moreover, the V_t_ decreased because the holes trapped in the states acted as local gates, and the J_T_ through the TFE increased due to the narrower depletion width at the source/drain Schottky interface. The V_t_ of the GaN SB-MOSFET without trap states was 1.7 V, regardless of the value of P_inc_. However, for conditions with donor-like trap states, the V_t_ shifted negatively to 1.5, 1.47, 1.4, and 0.6 V for the dark condition and P_inc_ had values of 0.001, 0.01, and 0.1 W/cm^2^, respectively, which were attributed to the PG effect.

Figure 10c displays the R_ph_ of the GaN SB-MOSFET according to the type of the trap state. In the absence of a trap state, the peak R_ph_ of the GaN SB-MOSFET at an incident wavelength of 360 nm was 0.012 A/W, although this increased to 0.021 A/W in the presence of donor-like trap states. Moreover, there was no change in the R_ph_ except around the wavelength of 365 nm, which corresponded to the cut-off wavelength of the GaN. This result was attributed to current leakage due to the PG effect. The PG effect was responsible for both the accumulation of positively ionized donor-like traps in Areas A and B and an increase in the I_dark_. The I_dark_ increased due to the depletion width of the thinned source/drain interface. In particular, as demonstrated in Figure 10a,d, the I_dark_ passing through Area B (where the trap states existed above the Fermi energy level) was considered to be quite high, resulting in a poor photo-current (I_photo_). Ultra-thin-body (UTB) MOSFETs and gate-all-around (GAA) transistors (such as vertical GaN nanowire MOSFETs and stacked nanosheet transistors) are fully depleted below the gate metal. Therefore, it is expected that a high R_ph_ value can be obtained by blocking the leakage path, although further study would be required to ensure compatibility with CMOS technology.

In the presence of the acceptor-like trap states, the R_ph_ of the GaN SB-MOSFET tended to decrease overall across the whole wavelength. This result was attributed to the significant decrease in the total current (I_dark_, I_photo_) due to the large Schottky barrier height and high channel resistance caused by the ionized acceptor-like traps in both Areas A and B. The peak R_ph_ of the GaN SB-MOSFET at 360 nm was 0.007 A/W, which decreased sharply as the trap concentration increased (not shown in the figure).

## 4. Conclusions

In this paper, we analyzed the effects of interface traps on the output characteristics of an inversion mode n-channel GaN Schottky barrier (SB)-MOSFET using a TCAD simulation. The I_off_ of the GaN SB-MSOFET with shallow donor-like trap states of (E_c_−0.06) and (E_c_−0.25) were 2.6 × 10^−10^ and 3.1 × 10^−13^ A/mm, respectively, indicating an increase compared with the value of 2.6 × 10^−21^ A/mm without trap states. The subthreshold swings (SSs) in this shallow trap state level were 358 and 227 mV/decade, respectively, which were considerably degraded compared to the no-trap condition with an SS of 65 mV/decade. In the presence of acceptor-like trap states at the SiO_2_/GaN interface, the I_on_ of the GaN SB-MOSFET at a V_GS_ of 5.0 V reduced to 2.32 mA/mm, which was attributed to the increased V_t_ and decreased tunneling current.

The I_off_ of the GaN SB-MOSFET with the donor-like trap states at the drain/GaN interface was 9.2 × 10^−19^ A/mm, indicating a significant increase compared with the value of 2.6 × 10^−21^ A/mm with donor-like trap states at the source/GaN interface. This result was attributed to a reduction in the resistive GaN path and the high tunneling rate at the drain/GaN interface. In the presence of the acceptor-like trap states at the drain/GaN interface, the V_DSsat_ and R_on_ increased considerably.

For the UV response, the photo-induced transfer characteristics of the GaN SB-MOSFET demonstrated that the photo-gating (PG) effect was the main mechanism causing the negative V_t_ shift and improved I_on_. The peak spectral responsivity due to the presence of shallow donor-like trap states considerably increased (near 365 nm) compared to the presence of the shallow acceptor-like trap states. In the presence of shallow acceptor-like trap states, the R_ph_ of the GaN SB-MOSFET tended to decrease over the whole wavelength.

For the improvement of the electrical performance of the GaN SB-MOSFET, high-quality GaN epitaxial growth, surface pre-treatment, and passivation technology are required to reduce the trap states. It is also necessary to identify the physical causality of the defects and interfacial trap states through GaN-based devices. We concluded that the shallow traps (such as V_N_) and the dislocation-related point defects shall be carefully reduced in the top epitaxial layer of the MOSFET. The simulation results demonstrated that the shallow trap affected the device’s switching performance and photo-response characteristics significantly, while the deep trap had a significant effect on the device’s on-state performance.

## Figures and Tables

**Table 1:** Device parameters for the reported and simulated GaN SB-MOSFETs.

Device Parameter	Reported Values [28]	Simulated Values
S-D Schottky barrier [eV]	0.2–0.3	0.3
SiO_2_ thickness [nm]	20	20
p-GaN carrier density [cm^−3^]	8.6 × 10^17^	2.7 × 10^17^
GaN thickness [μm]	0.7	0.7
Gate work function [eV]	5.0–5.1	5.0
Gate length [μm]	10	10
Gate width [μm]	30	30

**Table 2:** Summary of the interface trap states for c-plane p-type GaN used in the simulation study.

Position	Distribution	Nature	Origin	Trap Level from E_c_	Reported Density [cm^−3^]	Used Density in This Work [cm^−2^eV^−1^]
Metal/GaN interface (Trap Region A)	Discrete level	Donor	V_N_ *	0.06	—	2.1 × 10^17^ cm^−3^
			V_N_ or carbon-related	0.25	1.7×10^14^	7.0 × 10^17^ cm^−3^
		Acceptor	—	1.5	2.4×10^16^	7.0 × 10^17^ cm^−3^
			V_Ga_, V_Ga_-H complexes	2.6	2.6×10^16^	1.0 × 10^17^ cm^−3^
SiO_2_/GaN interface (Trap Region B)	Gaussian	Donor	V_N_	0.06	—	2.5 × 10^12^
			V_N_ or carbon-related	0.25	1.7×10^14^	4.0 × 10^12^
			C_I_ *	1.28	1.0×10^14^	4.2 × 10^11^
			C_I_	1.35	7.2×10^15^	7.8 × 10^11^
		Acceptor	V_Ga_ *, V_Ga_-H complexes	2.6	2.6×10^16^	1.5 × 10^11^
			Residual Mg_Ga_ and C acceptor	3.22	1.3×10^16^	2.5 × 10^11^
			C_N_ *	3.28	3.6×10^16^	4.0 × 10^11^

* V_N_ is the nitrogen vacancy, V_Ga_ is the gallium vacancy, C_I_ is the interstitial carbon, and C_N_ is the substitutional carbon.

## Data Availability

Additional data are available upon reasonable request.
